# Frailty and Quality of Life Among Community-Dwelling Older Adults

**DOI:** 10.7759/cureus.13049

**Published:** 2021-02-01

**Authors:** Ioanna V Papathanasiou, Anna Rammogianni, Dimitrios Papagiannis, Foteini Malli, Dimitrios C Mantzaris, Konstantinos Tsaras, Lamprini Kontopoulou, Evridiki Kaba, Martha Kelesi, Evangelos C Fradelos

**Affiliations:** 1 Faculty of Nursing, University of Thessaly, Larissa, GRC; 2 Refugees Reception Centre of Agia Varvara, National Organization of Public Health, Veroia, GRC; 3 Department of Nursing, University of West Attica, Athens, GRC; 4 Faculty of Nursing, University of Thessally, Larissa, GRC

**Keywords:** frailty, quality of life, aging, community healthcare, elderly people, health aging.

## Abstract

Older people often feel weak and have limited physical activity and walking capacity, without energy. These characteristics meet the conditions for the onset of the frailty syndrome. The effect that frailty syndrome can have on the elderly’s quality of life (QOL) status has not been sufficiently explored, especially in the Greek population. This study aims to investigate the correlation between frailty and QOL in a community population of elderly people with independent living. A descriptive cross-sectional study was performed. The study sample consisted of 257 elderly people from three Open Care Centers for the Elderly Population of the Municipality of Grevena, Greece. The Tilburg Frailty Indicator was used to measure frailty in elderly people and the World Health Organization QOL-BREF was used to assess the health-related QOL of older people. The majority of elderly people showed relatively low overall frailty score (mean: 5.44). The elderly people had relatively high QoL assessment values and general satisfaction with their health condition. The obtained results show a statistically significant negative relationship between (i) physical frailty, psychological frailty, and all dimensions of QOL, (ii) social frailty and social relationships, and (iii) total frailty and all dimensions of QOL. Consequently, despite an average age of 75.12 years and higher female participation, the study population was not very frail and were satisfied with their QOL. Frailty has a negative effect in all QOL domains.

## Introduction

Elderly people complain that they are weak and have limited physical activity and walking capacity. They feel exhausted, without energy, and weight loss is also often observed. These characteristics meet the conditions for the onset of the frailty syndrome. Although there have been many attempts to define the syndrome, the current literature has not reached a common definition yet [1]. Fried et al. defined frailty as a biological syndrome with detrimental effects on the body's systems, caused by limited resistance to stressors [2]. Other scientists describe it as a clinical syndrome that affects elderly people, as it may cause decreased functionality, increased falls, and probably increased hospitalization, disability, and mortality [3]. Frailty is a predisposition syndrome and its meaning is multidimensional. When the syndrome exists, a loss or reduction of some function, psychological, social, and physical, is observed. It is estimated that between 20% and 30% of seniors over the age of 75 years are vulnerable to frailty [4]*.*

The pathophysiology of the syndrome is not yet fully understood, but several body systems are involved [5]. The clinical syndrome of frailty is caused by biological, genetic, physical, environmental, social, and psychological factors [4]. Oxidative stress, which causes an inflammatory reaction and consequently an increase in white blood cells, has a role in the pathogenesis of the syndrome [1]. The syndrome may be prevented by proper nutrition, rich in nutrients and vitamins, by improving physical activity, and by reducing drug use. In addition, before the onset of the syndrome, there may be a pre-existing condition if two of the above factors are present [6].

Research has shown that women are more prone to the syndrome. Low socioeconomic status, education patterns, lifestyle (cigarette smoking, alcohol consumption), psychological state, such as the presence of depressive symptoms, and disability may also be risk factors for frailty [4]. The frailty phenotype is based on a set of five criteria mentioned previously, while the frailty scale is based on a series of health deficits of the elderly. Other frailty scales than the one developed by Fried et al. show a better ability to predict mortality [7]. According to the literature, women are more vulnerable to frailty perhaps because of their lower weights and less fat mass [3]. Research based on the study conducted by Fried et al. [2] showed that frailty prevalence is 7-12% in adults of 65 years or older in the United States. This prevalence increases by 3.9% in those aged 74 years and older and by almost 25% in those aged 85 years and older. The syndrome is more common in women. Africans (13%) appear more vulnerable than Caucasians (6%) when facing risk for frailty. With regard to Europe, older persons in Spain have the highest prevalence (27%), while those in Switzerland have the lowest prevalence (5.8%) [8]. Frailty prevalence in Latin American ranges from 30% to 48% in women and from 21% to 35% in men [1]. According to a study of Ntanasi et al. [9], the prevalence of frailty ranged between 4.1% and 24.5% when using the Fried et al. definition and the Tilburg Frailty Indicator (TFI) instrument to measure frailty, respectively [9]. Some research suggests that clinical syndrome is present in all older people over the age of 95 years old [4]. A study which involved 1,867 older people found that participants aged 80 years or older had almost 13 times higher odds of being frail [9]. The prevalence of frailty is 3.2%, 16.3% and 23.1% for those aged 65, over 80, and over 90 years, respectively [10].

Quality of life (QOL), according to the World Health Organization, includes each person's sense of life related to values, culture, standards, interests, and goals in his life. Therefore, quality of life is subjectively expressed in the form of an individual's pleasure and satisfaction with life. It is influenced by an individual's physical and mental health and condition as well as by human relationships [11]. Elderly people have a better quality of life when they have high self-esteem, are adaptable and shape their lifestyle according to their own needs, when they are able to take care of themselves, optimistic and satisfied with their daily lives and social relationships [12].

Research showed that quality of life is rated higher in people aged over 50 to 68 years and later tends to decline significantly after 86 years old. Also, for people over the age of 80, physical health, functionality and independence are more important for their quality of life [13]. Sharma et al. [13] conducted a study with 129 elderly people whose mean age was 68.44 years. 87% of the participants were found to have cognitive deficits, which are negatively associated with physical function and therefore the quality of life. In everyday life, cognitive functioning is especially important for decision-making, goal setting, work planning, and problem-solving, and therefore, for the independence of the individual. It is also concluded that elderly people with more years of education have reduced cognitive impairment, better physical and family environment, and better living standards and nutrition [13]. In addition, elderly people who build and maintain social interactions with younger people are more optimistic and have a better quality of life. They have increased mental resilience due to their support network. For people of different cultural backgrounds, the distance from their country of birth and from the family environment can be an important indicator of decreased quality of life [14].

Physical activity can affect frailty syndrome, as it is associated with elderly people's quality of life improvement and can help to maintain and improve balance, avoid falls, lower your blood sugar levels and stress [15]. Women, especially over 80 years of age, are more vulnerable while elderly people who maintain their social relationships and their physical condition have a better quality of life compared to those who suffer from chronic diseases or live alone [15].

The aim of this study is to investigate the correlation between frailty and quality of life among a community population of elderly people with independent living.

## Materials and methods

A descriptive cross-sectional study was performed. The study sample consisted of elderly people who were recipients of the services and active members of Open Care Centers for the Elderly Population (KAPI) of the Municipality of Grevena. The sampling method used was that of convenience sampling. We included a total sample of 257 elderly people from three Open Care Centers for the Elderly Adults. Inclusion criteria were as follows:

A. People aged ˃60 years.

B. Active members of Open Care Centers for the Elderly (KAPI) of the Municipality of Grevena.

C. Self-care ability and independent living ability.

D. Persons without mental or cognitive impairment.

E. Knowledge of the Greek Language and ease of communication.

F. Consent to participate in research

Data collection was carried out using a fully structured questionnaire consisting of three parts as follows:

A. A form of individual characteristics of the elderly people.

B. Included questions about their socio-demographic characteristics (gender, age, marital status, number of children, level of education, area of ​​permanent residence, individual's monthly income) and the characteristics regarding self-assessment of health status and living (living alone or with others, healthy lifestyle, presence of chronic diseases or disorders, experiencing psychologically charged situations, satisfaction with the family environment).

C. The Tilburg Frailty Indicator was used to measure frailty in the elderly people [16]. The TFI is a self-report questionnaire to detect frailty in elderly people and is made up of a total of 15 questions related to the assessment of three sub-dimensions of frailty: physical domain of frailty, which consists eight components; psychological domain of frailty, which consists of four components; and social domain of frailty, which consists three domains. Eleven questions from the TFI have two response categories -- ‘‘yes’’ and ‘‘no’’ -- and four questions have three response categories -- "yes", "sometimes", and "no". Responses are scored with values from 0 to 1. The total score of the scale and the subscales is calculated by the sum of the answers and a higher score indicates a higher level of frailty. According to the authors, the TFI has satisfactory psychometric properties in terms of reliability and validity in samples of elderly people living in the community [16]. A Greek version of TIF, that was culturally adapted, was used in the current study.

D. The Greek version of the World Health Organization Quality of Life - BREF (WHOQOL-BREF) was used to assess the health-related quality of life of elderly people [17]. The full-length WHOQOL was originally developed in 1994 by the World Health Organization aiming to develop an integrated QOL measurement tool suitable for cross-cultural comparison in diseased and healthy individuals. The short version, called WHOQOL-BREF, which was used in the present study, was translated and adjusted in Greek by Ginieri-Coccossis et al. [17]. The results of the psychometric properties of the WHOQOL-BREF Greek version showed that the questionnaire had a satisfactory level of internal consistency reliability, test-retest reliability, construct validity, convergent validity, and discriminant validity. According to the authors, the questionnaire is a reliable and valid instrument for assessing the quality of life and can be used in the wider field of health care to assess the quality of life of clinical groups and healthy individuals. The Greek version of the WHOQOL‐BREF questionnaire consists of 30 questions, of which four sub-scales are obtained: nine questions refer to "Physical Health", six questions refer to "Psychological Health", five questions refer to “Social Relationships”, and eight questions refer to “Environment”. The first two questions refer to the overall quality of life in general as well as to the degree of satisfaction with the state of health and constitute the domain “global quality of life/general health”. Responses are given on a 5-point Likert scale ranging from 1 (very poor or not at all or very dissatisfied or never) to 5 (very good or too much or too satisfied or continuously). Three questions are reverse scored. The total score of each domain is calculated from the average of the sum of the questions constituting each subscale, multiplied by 4. Thus, total scores for each domain range from 4 to 20. Higher scores indicate a higher quality of life in each domain.

Data collection was carried out at the three KAPI of the Municipality of Grevena during the period of March to April 2019 after obtaining permission from the competent municipality office to proceed with the survey. The completion of the questionnaires was done by the participants themselves after informing them about the purpose of the research. All necessary clarifications were provided. The participation of the elderly people was voluntary and in accordance with the principles and ethical rules of research.

Statistical analysis was performed with SPSS Version 22.0 statistic software package for Windows (IBM Corp., Armonk, NY, USA) to obtain descriptive and inferential statistics. In particular, the descriptive analysis included the frequency distribution for qualitative variables (absolute and relative % frequency) and estimates of position and dispersion parameters of the quantitative variables (mean, median, standard deviation, minimum and maximum value). To investigate possible correlations, we used Pearson's correlation coefficient. A multiple linear regression analysis was conducted to explore relationships between TFI subscales and WHOQOL-BREF dimensions, after adjusting to individual characteristics of elderly people. All reported p-values were two-tailed, and the statistical significance level was set at 0.05.

## Results

The sample of the present study consisted of 114 men (44.4%) and 143 women (55.6%). Their ages ranged from 61 to 96 years while the mean age was 75.12 years. 65.4% were married and 30.4% widowed. Three-fifths of the participants had 1 to 2 children (60.7%). Most of them were primary school graduates (68.5%) and lived in a non-urban area (54.9%). Regarding their individual monthly income, 55.6% of participants had an income ranging from 500 to 1,000 euros. About 3/4 of the elderly lived with family members or others (75.9%). 61.9% described their lifestyle as healthy and 54.9% did not have two or more chronic diseases or disorders. Regarding experiencing psychological problems, 27.2% had experienced the death of loved one, 19.1% a serious illness, 23.0% serious illness of a loved one, 7.0% a divorce or the end of an important relationship, 0.8% a car accident, and 2.3% criminal activity. Finally, the vast majority of the elderly (93.0%) reported that they were satisfied with their family environment (Table 1).

**Table 1:** Individual characteristics of older people (n = 257)

Characteristics	n	%
Gender		
Male	114	44.4
Female	143	55.6
Age (years)		
Mean ± SD	75.12 ± 8.39	
Min – Max	61 – 96	
Marital status		
Single	4	1.6
Married	168	65.4
Divorced	7	2.7
Widowed	78	30.4
Number of children		
0	8	3.1
1 – 2	156	60.7
≥ 3	93	36.2
Education level		
Primary education	176	68.5
Secondary education	61	23.7
Tertiary education	20	7.8
Permanent residence area		
Urban	116	45.1
Non-urban	141	54.9
Individual monthly income (Euro)		
< 500	63	24.5
500 – 1.000	143	55.6
˃ 1.000	51	19.8
Cohabitation		
Alone	62	24.1
Family members or others	195	75.9
How would you describe your lifestyle?		
Healthy	159	61.9
Neither healthy nor unhealthy	90	35.0
Unhealthy	8	3.1
Do you have two or more chronic diseases or disorders?		
Yes	116	45.1
No	141	54.9
Did you experience any of the following during the previous year?		
Death of a loved one	70	27.2
Serious disease	49	19.1
Serious illness of a loved one	59	23.0
Divorce or the end of an important relationship	18	7.0
Car accident	2	0.8
Criminal action against you	6	2.3
Are you satisfied with your family environment?		
Yes	239	93.0
No	18	7.0

Internal consistency reliability, which was determined through the Cronbach's alpha coefficient of the total frailty, was α = 0.75, while internal consistency reliability of the three subscales was α = 0.74 for the physical domain, α = 0.68 for the psychological domain, and α = 0.21 for the social domain, which is considered as very low. TFI subscales showed very good internal consistency reliability, with the exception of the social domain of frailty, which had low internal consistency reliability (Table 2). The overall frailty score ranged from 0 to 13, with a mean of 5.44 (standard deviation = 3.02) and median value 5 (Table 2). According to the cut-off point (4/5), the TFI indicated 139 participants with frailty (54.1%) and 118 without frailty (45.9%).

**Table 2:** Tilburg Frailty Indicator of the elderly

Tilburg Frailty Indicator (TFI)	Cronbach’s alpha	Mean ± SD	Median	Min – Max
Physical Frailty	0.74	2.70 ± 2.16	2.00	0 – 8
Psychological Frailty	0.68	1.43 ± 1.21	1.00	0 – 4
Social Frailty	0.21	1.32 ± 0.64	1.00	0 – 3
Total Frailty	0.75	5.44 ± 3.02	5.00	0 – 13

Internal consistency reliability, shown by Cronbach's alpha coefficient, for the WHOQOL-BREF was α = 0.87; for the physical health domain α = 0.84, for the psychological health domain α = 0.73, for the social relationships domain α = 0.70, for the environment domain α = 0.68, and for global quality of life/general health α > 0.70, indicating a very good internal consistency reliability score for the scale (Table 3). The global quality of life/general health score ranged from 4.00 to 20.00 with a mean of 14.14 (standard deviation = 2.87) and median value 14.00. The median value was greater than the midpoint of the measurement scale of the responses, i.e., the value 12, indicating that the elderly population had a relatively high quality of life assessment values and general satisfaction with their state of health (Table 3).

**Table 3:** WHOQOL-BREF among elderly (n = 257). WHOQOL-BREF: World Health Organization Quality of Life - BREF

WHOQOL-BREF	Cronbach’s alpha	Mean ± SD	Median	Min – Max
Physical Health	0.87	13.56 ± 2.79	13.78	6.22 – 20.00
Psychological Health	0.84	13.61 ± 2.74	14.00	4.67 – 20.00
Social Relationships	0.73	13.72 ± 2.60	13.60	4.80 – 20.00
Environment	0.70	13.70 ± 1.96	14.00	8.00 – 20.00
Global quality of life/general health	0.68	14.14 ± 2.87	14.00	4.00 – 20.00

Table 4 presents data on the correlation between TFI and the WHOQOL-BREF scales of the elderly.

**Table 4:** Correlation of TFI with WHOQOL-BREF Scale Data presented are Pearson's correlation coefficient (r) *p<0.05 **p<0.01 ***p<0.001 WHOQOL-BREF: World Health Organization Quality of Life - BREF; TFI: Tilburg Frailty Indicator

TFI scale	WHOQOL-BREF scale				
	Physical Health	Psychological Health	Social Relationships	Environment	Global Quality of Life/General Health
Physical Frailty	-0.752^***^	-0.586^***^	-0.481^***^	-0.331^***^	-0.438^***^
Psychological Frailty	-0.533^***^	-0.568^***^	-0.465^***^	-0.370^***^	-0.419^***^
Social Frailty	-0.031	-0.032	-0.220^***^	-0.034	-0.041
Total Frailty	-0.757^***^	-0.653^***^	-0.577^***^	-0.392^***^	-0.490^***^

The statistical analysis revealed the following main results:

A statistically significant negative relationship between physical frailty and all dimensions of quality of life among elders was found. In particular, when physical frailty levels decrease, elderly people's QOL regarding physical health (r = -0.752), psychological health (r = -0.586), social relationships (r = -0.481), environment (r = -0.331), and global quality of life/general health (r = -0.438) increases.

A statistically significant negative relationship between psychological frailty and all dimensions of QOL among elderly people was found. In particular, when psychological frailty levels decrease, elderly people's QOL regarding physical health (r = -0.533), psychological health (r = -0.568), social relationships (r = -0.465), environment (r = -0.370), and global quality of life/general health (r = -0.419) increases.

A statistically significant negative relationship between social frailty and social relationships dimension of QOL among elders was found. In particular, when social frailty levels decrease, elderly people’s QOL regarding social relationships (r = -0.220) increases.

Finally, a statistically significant negative relationship between total frailty and all dimensions of quality of life among elders was found. In particular, when total frailty levels decrease, elderly people’s QOL regarding physical health (r = -0.757), psychological health (r = -0.653), social relationships (r = -0.577), environment (r = -0.392), and global quality of life/general health (r = -0.490) increases.

A multiple linear regression analysis was performed to explore the relationship between frailty and quality of life among older people. Table 5 presents the regression coefficients (β) for TFI subscales scores in WHOQOL-BREF domains scores after controlling for sociodemographic and other characteristics. The results showed a negative impact of frailty on the elderly's quality of life, except the “social frailty” subscale.

**Table 5:** Multiple regression results with WHOQOL-BREF dimensions as dependent variables and TFI subscales as independent, adjusted for individual characteristics of older people. ^a ^Regression coefficient (standard error) adjusted for socio-demographic and other characteristics. *p<0.05 **p<0.01 ***p<0.001 WHOQOL-BREF: World Health Organization Quality of Life - BREF; TFI: Tilburg Frailty Indicator

TFI scale	WHOQOL-BREF scale				
	Physical Health	Psychological Health	Social Relationships	Environment	Global Quality of Life/General Health
	β (SE)^a^	β (SE)^a^	β (SE)^a^	β (SE)^a^	β (SE)^a^
Physical Frailty	-0.749^***^ (0.063)	-0.524^***^ (0.077)	-0.379^***^ (0.074)	-0.205^**^ (0.063)	-0.525^***^ (0.082)
Psychological Frailty	-0.776^***^ (0.115)	-0.902^***^ (0.119)	-0.590^***^ (0.117)	-0.396^***^ (0.099)	-0.686^***^ (0.133)
Social Frailty	-0.176 (0.323)	0.216 (0.340)	-0.436 (0.317)	-0.055 (0.263)	0.093 (0.361)
Total Frailty	-0.577^***^ (0.045)	-0.470^***^ (0.053)	-0.346^***^ (0.053)	-0.198^***^ (0.046)	-0.428^***^ (0.059)

Physical frailty has a statistically significant negative effect on the domains of QOL. More specifically, on physical health (β = -0.749, p < 0.001), psychological health (β = -0.524, p < 0.001), social relationships (β = -0.379, p ˂ 0.001), environment (β = -0.205, p ˂ 0.01), and overall quality of life/general health (β = -0.525, p ˂ 0.001). On the other hand, psychological frailty had also a negative effect on physical health (β = -0.776, p < 0.001), psychological health (β = -0.902, p < 0.001), social relationships (β = -0.590, p ˂ 0.001), environment (β = -0.396, p ˂ 0.01), and overall quality of life/general health (β = -0.686, p ˂ 0.001). Finally, total frailty was an independent predicting factor for the quality of life associated with physical health (β = -0.577, p < 0.001), psychological health (β = -0.470, p < 0.001), social relationships (β = -0.346, p ˂ 0.001), environment (β = -0.198, p ˂ 0.01), and overall quality of life/general health (β = -0.428, p ˂ 0.001).

## Discussion

The Greek population is aging rapidly, especially the population of small provincial prefectures and municipalities such as Grevena. It is a global phenomenon which is particularly caused in developed countries in the Western world. This leads scientists to further engage and conduct research to address issues relating to the aging population. The prevalence of physical frailty was higher than the one of psychological and social frailty, as most suffer from one or more chronic diseases. The overall frailty score among the elderly in the municipality of Grevena appears to be reduced. Regarding the quality of life of the elderly people it was found that they are quite satisfied with their social relations at first, then with the environment and then with their psychological and physical health. Regarding the global quality of life/general health, it is concluded that they are also satisfied with the state of their life and its quality. When investigating the relationship between frailty and quality of life, the results of the multiple regression showed negative correlations, also statistically significant, between all dimensions of the two scales, except the dimension of social frailty and its relations with the dimensions of QOL.

One possible reason for low frailty levels and high QOL levels compared to other studies is maybe due to the small sample size. However, in the present study, an important relationship between frailty syndrome and quality of life was found. In a study by Langlois et al. [18], older adults' (aged 61 to 89 years) quality of life was found to have a significant negative relationship with all dimensions of frailty [18]. In another study conducted with the participation of 374 elderly people and using Fried frailty criteria to identify frailty and health-related QOL (HRQOL) questionnaire to measure participants' quality of life, frail participants had a reduced quality of life, compared to those who were pre-frail and who exhibited low SF-36 scores in only the mental component scale [19].

Uchmanowicz and Gobbens [20] conducted a study with a sample of 100 patients with heart failure, using the TFI and HRQOL questionnaire. Frailty was found in 89% of the studied population while frailty was found to have a significantly negative impact on the health-related quality of life results. There was a greater negative relationship between psychological frailty and mental health QOL [20], while in the present study, physical and psychological frailty have the greatest negative relationship with QOL. A study by Bagshaw et al [21], involved 421 people over the age of 50, and used EuroQol Health Questionnaire and the SF-12. 33% of participants were frail while the older the population, the higher the level of frailty and the lower the quality of life [21]. In addition, the results of the present study and those of Ntanasi et al. [9] agreed about the increasing rates in old age, with female sex, low socioeconomic status and educational background, and those without a partner being most vulnerable [9].

Other studies showed that quality of life increases in middle-aged people and decreases in elderly people (after 70-80 years). In the results of such studies exploring conditions associated with elderly people, it is necessary to take into consideration the awareness of old age, past life experiences, abilities and achievements, which help the elderly feel fullness and satisfaction with their lives [22-25].

The limitations of the study include the lack of time margins as the questionnaires were completed within two months (March-April 2019). Also, the elderly population of the municipality of Grevena, which is member of Open Care Centers for the Elderly Population is not very large, so the study sample consists of 257 people and the selection of the participants was done through convenience sampling. Many elderly people experienced difficulties in completing the questionnaire because of health problems or low educational level. Many of them refused to participate in the completion of the questionnaire. Results from this study, therefore, derived exclusively from members of Open Care Centers for the Elderly Population and not from the general elderly population of the municipality of Grevena. Another limitation is also the existence of many different tools with different criteria and characteristics for the frailty syndrome. Also, studies with a long duration and a bigger population are necessary to be conducted. The above limitations of the present study create the need for new research studies in larger samples, with random sampling for greater validity of results, and the future use of a common definition and tool for frailty syndrome for an optimal verification result. This will make it easier to draw conclusions concerning frailty syndrome and quality of life so that plans can be made to help improve elderly people’ lives.

## Conclusions

In conclusion, we found that despite the average age of older people which was 75.12 years (61-96), and higher female participation, participants in the study were not very frail and were satisfied with their quality of life. This study examined the relationship between frailty and quality of life and provides information about the proportion of frailty and the levels of QOL among community-dwelling older adults, specifically among those who live independently and are active members of community centers for the elderly. The proportion of frailty was found to be rather high 54.1% and participants reported a good level of QOL. Finally, frailty can have a negative impact on QOL of community-dwelling adults.

However, the conduction of new studies with a larger sample size in the Greek region will bring more reliable and more valid results. Moreover, the need to provide new information to healthcare professionals will lead to faster information transmission, awareness and education of them regarding geriatric patients, and will guide them to improve their healthcare and their lives in general.
